# Characterization of two near-infrared genetically encoded voltage indicators

**DOI:** 10.1117/1.NPh.11.2.024201

**Published:** 2023-12-11

**Authors:** Chenchen Song, Mikhail E. Matlashov, Daria M. Shcherbakova, Srdjan D. Antic, Vladislav V. Verkhusha, Thomas Knöpfel

**Affiliations:** a Imperial College, Laboratory for Neuronal Circuit Dynamics, London, United Kingdom; b Nanyang Technological University, Singapore; c Albert Einstein College of Medicine, Gruss-Lipper Biophotonics Center, Department of Genetics, Bronx, New York, United States; d Institute for Systems Genomics, UConn Health, Department of Neuroscience, Farmington, Connecticut, United States; e University of Helsinki, Medicum, Faculty of Medicine, Helsinki, Finland; f Hong Kong Baptist University, Laboratory for Neuronal Circuit Dynamics, Hong Kong, China

**Keywords:** genetically encoded voltage indicator, voltage imaging, wide-field optical imaging, opsin, voltage-sensing domain, iRFP

## Abstract

**Significance:**

Efforts starting more than 20 years ago led to increasingly well performing genetically encoded voltage indicators (GEVIs) for optical imaging at wavelengths <600  nm. Although optical imaging in the >600  nm wavelength range has many advantages over shorter wavelength approaches for mesoscopic *in vivo* monitoring of neuronal activity in the mammalian brain, the availability and evaluation of well performing near-infrared GEVIs are still limited.

**Aim:**

Here, we characterized two recent near-infrared GEVIs, Archon1 and nirButterfly, to support interested tool users in selecting a suitable near-infrared GEVI for their specific research question requirements.

**Approach:**

We characterized side-by-side the brightness, sensitivity, and kinetics of both near-infrared GEVIs in a setting focused on population imaging.

**Results:**

We found that nirButterfly shows seven-fold higher brightness than Archon1 under the same conditions and faster kinetics than Archon1 for population imaging without cellular resolution. But Archon1 showed larger signals than nirButterfly.

**Conclusions:**

Neither GEVI characterized here surpasses in all three key parameters (brightness, kinetics, and sensitivity), so there is no unequivocal preference for one of the two. Our side-by-side characterization presented here provides new information for future *in vitro* and *ex vivo* experimental designs.

## 1 Introduction

Concerted efforts in the development of neurotechnologies and molecular tools over the past 5 years have rapidly expanded the toolkit for monitoring neuronal activity.^1^^,^^2^ One of those tools is genetically encoded voltage indicators (GEVIs), a long sought-after means for real-time reporting of the electrical activities of excitable cells.^3^

Fully genetically encoded GEVIs stem from two main families of molecular scaffolds. Voltage sensing domain (VSD) based GEVIs arise from fusing one or a pair of fluorescent proteins onto a VSD from *Ciona intestinalis*^4^^–^^8^ or *Gallus gallus*.^9^^–^^13^ As an alternative design, opsin-based GEVIs capitalize on the voltage sensitivity of microbial opsins and report changes in membrane potential either as intensity changes of the native opsin fluorescence^14^^–^^17^ or of the voltage-dependent quenching of an attached bright fluorescent protein.^18^^–^^20^

**Fig. 1:**
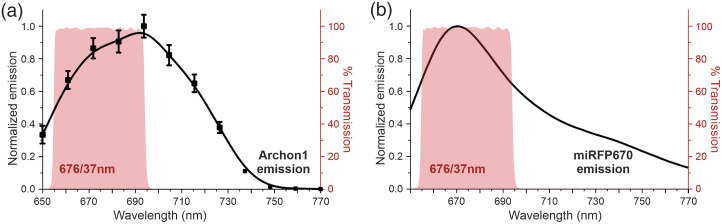
Archon1 and nirButterfly features. (a) Structural schematic of Archon1. (b) The normalized emission spectrum of Archon1 (N=3 cells, mean ± SEM). The red shaded area denotes the transmission band of the 676/37 nm emission filter used for the epifluorescence side-by-side functional comparison experiments. (c) Structural schematic of nirButterfly. (d) Same as in (b), with the normalized emission spectrum of miRFP670,^22^ the donor fluorescent protein of nirButterfly.

Efforts to develop both GEVI families, VSD-based and opsin-based, led to variants with increasingly better performance properties, particularly extending the available GEVI spectral palette into the near-infrared range. Optical imaging in the near-infrared portion of the light spectrum has several methodological advantages, particularly for application in the mammalian brain:^21^ (1) reduced influence of hemodynamic components in the GEVI optical signal, (2) reduced tissue scattering, and (3) increased tissue penetrance. These benefits fueled efforts that generated two top-performing near-infrared GEVIs: a seven transmembrane domain Archon1 [Fig. 1(a)] of the opsin-based family^15^ and a four transmembrane domain nirButterfly [Fig. 1(c)] of the VSD-based structural design family.^23^ Here, we performed a side-by-side comparison of these two near-infrared GEVIs. As with many tools, there is no one-size-fits-all solution; therefore, we focus on *ex vivo* wide-field imaging and present our findings, so tool users may decide on the trade-offs for the desirable qualities required for their specific experiments.

## 2 Methods

### 2.1 Animals

All procedures were performed following the UK Animal Scientific Procedures Act (1986) under Home Office Personal and Project licenses following appropriate ethical review.

### 2.2 Cloning and AAV Production

To generate the pCAG-nirButterfly-IRES-EGFP vector, the CAG promoter fragment amplified from pCAG-Kir2.1-T2A-tdTomato (Addgene #98804), the nirButterfly fragment amplified from pCAG-nirButterfly (Addgene #136590), and the IRES2-EGFP fragment amplified from pIRES2-EGFP (Clontech) were subcloned into pcDNA3.1/Hygro(+) (Invitrogen). The pCAG-Archon1-IRES-EGFP vector was generated by subcloning the Archon1 fragment amplified from the pAAV-CaMKII-Archon1-EGFP vector (Addgene #108417) into the pCAG-nirButterfly-IRES-EGFP vector to replace the nirButterfly coding sequence. AAV vector plasmids, pAAV-CaMK2A-nirButterfly and pAAV-CaMK2A-Archon1, were produced by subcloning nirButterfly and Archon1, respectively, into the pAAV-CW3SL vector in place of EGFP (Addgene #61463) to express the GEVIs under identical virus plasmid backbones. The AAV plasmids were separately packaged into the AAV1 capsid by Penn Vector Core to produce AAV1.CaMK2A.nirButterfly or AAV1.CaMK2A.Archon1 at similar virus titers.

### 2.3 Cell Culture, Plasmid Transfection, and AAV Transfection

#### 2.3.1 Plasmid transfection of HEK293T cells

HEK293T cells were maintained in DMEM-GlutaMax (Life Technologies) supplemented with 5% FBS (Life Technologies). Transfections were performed using Lipofectamine 2000 (Life Technologies). Cells were transfected with either pCAG-nirButterfly-IRES-EGFP or pCAG-Archon1-IRES-EGFP, without biliverdin or all-trans-retinal (for GEVI comparison between nirButterfly and Archon in HEK cells). Cells were re-plated onto poly-D-lysine-coated coverslips at least 8 h after transfection.

#### 2.3.2 AAV transduction of culture primary neurons

Primary cultures of cortical and hippocampal neurons were dissociated from P0.5 C57Bl6 mice, plated onto poly-D-lysine coated coverslips, and maintained as per published protocols.^23^^,^^24^

Cells were transduced with AAVs 4 days after plating. The same batches of primary neuronal cultures at similar plating densities were side-by-side transduced with either AAV1.CaMK2A.nirButterfly or AAV1.CaMK2A.Archon1 under identical virus plasmid backbones (packaged by Penn Vector Core) at 1e8 vg per 1e5 cells. At DIV 10, a subset of neurons transduced with either AAV1.CaMK.nirButterfly or AAV1.CaMK.Archon1 was additionally supplemented with their respective chromophores biliverdin (1  μM, Sigma) or all-trans-retinal (1  μM, Sigma). Neurons were used for confocal imaging at 24 h later with media refreshed at least 5 h prior to imaging.

### 2.4 Confocal Imaging

Transduced cultures were expressed for at least 1 week before imaging.

#### 2.4.1 Measuring the fluorescence emission spectrum of Archon1

To measure the emission spectrum of Archon1, live primary neuronal cultures were transduced with AAV1.CaMK.Archon1 and imaged using a Leica TCS SP8 confocal microscope equipped with a 20× (NA1.40) objective using a 633 nm He–Ne laser for Archon1 excitation, and emission lambda stacks were acquired.

#### 2.4.2 Side-by-side comparison of GEVI brightness in primary cultured neurons

For side-by-side brightness comparisons of Archon1 and nirButterfly, the same batches of live primary neuronal cultures were transduced with either AAV1.CaMK2A.nirButterfly or AAV1.CaMK2A.Archon1 and imaged using a Leica TCS SP8 confocal microscope equipped with a 10× (NA0.4) air objective and a 63× (NA1.40) oil immersion objective.

Images were acquired under identical imaging conditions, including a 633 nm He–Ne laser light of identical intensity that was used for GEVI excitation, GEVI emission recorded over a similar wavelength range, and with similar acquisition settings, such as dwell and scanning time and pin-hole size.

### 2.5 Surgery

For performance comparison experiments between nirButterfly and Archon1 in acute brain slices, C57Bl6 mice aged 21 to 22 days were used (N=6, either sex). All mice underwent isoflurane-induced surgical anesthesia induced at 5% isoflurane carried in oxygen and maintained at 1% isoflurane in oxygen. To transduce the hippocampus with either nirButterfly or Archon1, the skull over the somatosensory cortex was gently thinned, and the pipette was used to puncture through the thinned skull to deliver 1e10 vg of either AAV1.CaMK2A.nirButterfly or AAV1.CaMK2A.Archon1 (Penn Vector Core USA) into hippocampal CA1 or the cortex over three depths at the rate of 0.2  nl/s (Nanoject II, Drummond). The pipette was left at the final depth for an additional 5 min before being retracted, and skin over the scalp was sutured.

### 2.6 Population Imaging in Acute Brain Slices

Acute brain slices were prepared from AAV-injected animals after 3 weeks of expression time. All mice were terminally anesthetized (ketamine/xylazine ip), transcardially perfused with 10 ml of ice-cold sucrose-based cutting solution (in mM: 0.3 KCl, 2.6 ${\mathrm{NaHCO}}_{3}$, 0.125 ${\mathrm{NaH}}_{2}{\mathrm{PO}}_{4}$, 0.3 sodium pyruvate, 190.0 sucrose, 25.0 dextrose, 0.5 ${\mathrm{CaCl}}_{2}$, 4.0 ${\mathrm{MgCl}}_{2}$), and then decapitated, and brains were removed under immersion in ice-cold cuttings solution that bubbled with $95\%O_{2}$ to $5\%{\mathrm{CO}}_{2}$. Coronal sections (300  μm thickness) were cut with a vibratome (Leica VT1200S, Leica Microsystems) and transferred into holding artificial cerebrospinal fluid (in mM: 12.6 NaCl, 3.5 KCl, 26 ${\mathrm{NaHCO}}_{3}$, 0.125 ${\mathrm{NaH}}_{2}{\mathrm{PO}}_{4}$, 1.0 dextrose, 2.0 ${\mathrm{CaCl}}_{2}$, 2.0 ${\mathrm{MgSO}}_{4}$) bubbled with 95% $O_{2}$ TO $5\%{\mathrm{CO}}_{2}$. Slices were first held at 33°C for 30 min and then transferred to room temperature for at least another 30 min recovery. For recordings, single slices were transferred into the recording chamber mounted on the stage of a dual emission widefield epifluorescence immersion microscope (Scientifica, United Kingdom). Slices in the recording chamber were superfused with recording ACSF bubbled with $95\%O_{2}$ to $5\%{\mathrm{CO}}_{2}$ (in mM: 12.6 NaCl, 3.5 KCl, 26 ${\mathrm{NaHCO}}_{3}$, 0.125 ${\mathrm{NaH}}_{2}{\mathrm{PO}}_{4}$, 1.0 dextrose, 1.2 ${\mathrm{CaCl}}_{2}$, 1.0 ${\mathrm{MgSO}}_{4}$) at 32°C to 34°C.

For synaptic stimulation, borosilicate glass pipettes were pulled on a two-stage vertical puller (PC-10, Narishige, Japan) to a pipette resistance of 0.5 to 1  MΩ and filled using the recording ACSF (in mM: 12.6 NaCl, 3.5 KCl, 26 ${\mathrm{NaHCO}}_{3}$, 0.125 ${\mathrm{NaH}}_{2}{\mathrm{PO}}_{4}$, 1.0 Dextrose, 1.2 ${\mathrm{CaCl}}_{2}$, 1.0 ${\mathrm{MgSO}}_{4}$). For the Gabazine condition, 5  μM Gabazine (Tocris) was added to the recording ACSF, and the slice was superfused with Gabazine-ACSF for 10 min before recording. For the Gabazine recovery condition, the slice was superfused with the original recording ACSF for 30 min before recording.

The epifluorescence immersion microscope was equipped with a CMOS camera (acA1920-155  μm, BaslerAG) or photodiode (TILL Photonics, Gräfelfing, Germany), using LED light sources (CoolLED, United Kingdom; FiberOptoMeter, NPI Electronic, Germany) and with the following optical filters: (Semrock and Chroma): miRFP670 (donor) excitation FF02-615/29, excitation beamsplitter 635LP (FF635-Di01), and miRFP670 emission 676/37. Signals were acquired with an Axon 700B Multiclamp amplifier and digitized at 10 kHz with a Digidata 1440A using pCLAMP software (Molecular Devices, San Jose, California) for data acquisition and the synchronization of stimulus delivery. Only the donor channel was recorded for nirButterfly. Identical optical filters were used for functional imaging experiments with Archon1.

### 2.7 Data Analysis

Spectral and intensity data were analyzed post-hoc using Leica Application Suite X.

Manual ROIs for optical traces in functional imaging experiments were generated in ImagePro. Fluorescence intensity quantifications for GEVI brightness comparison were performed using ImagePro.

The quantification of photodiode recordings and the fitting of response time constants were analyzed using custom MATLAB scripts from the Knopfel Lab MATLAB scripts library.

All statistical tests are performed using MATLAB.

## 3 Results

First, we wanted to confirm that our optical configuration for nirButterfly detection is sufficient for detecting the fluorescence emission from Archon1. Archon1 emission spectra were not documented, so we measured the fluorescence emission spectrum of Archon1-expressing HEK293T cells, using 638 nm laser excitation under lambda stack confocal configuration [Fig. 1(b)].

These experiments confirmed that the emission detection used in our imaging setup transmits a large and comparable fraction of the emitted fluorescence of both nirButterfly and Archon1. As both near-infrared GEVIs have similar spectral properties [Figs. 1(b) and 1(d)], we could directly compare three GEVI key features: brightness, sensitivity, and kinetic properties.

### 3.1 nirButterfly Exhibits Sevenfold Higher Brightness than Archon1

**Fig. 2:**
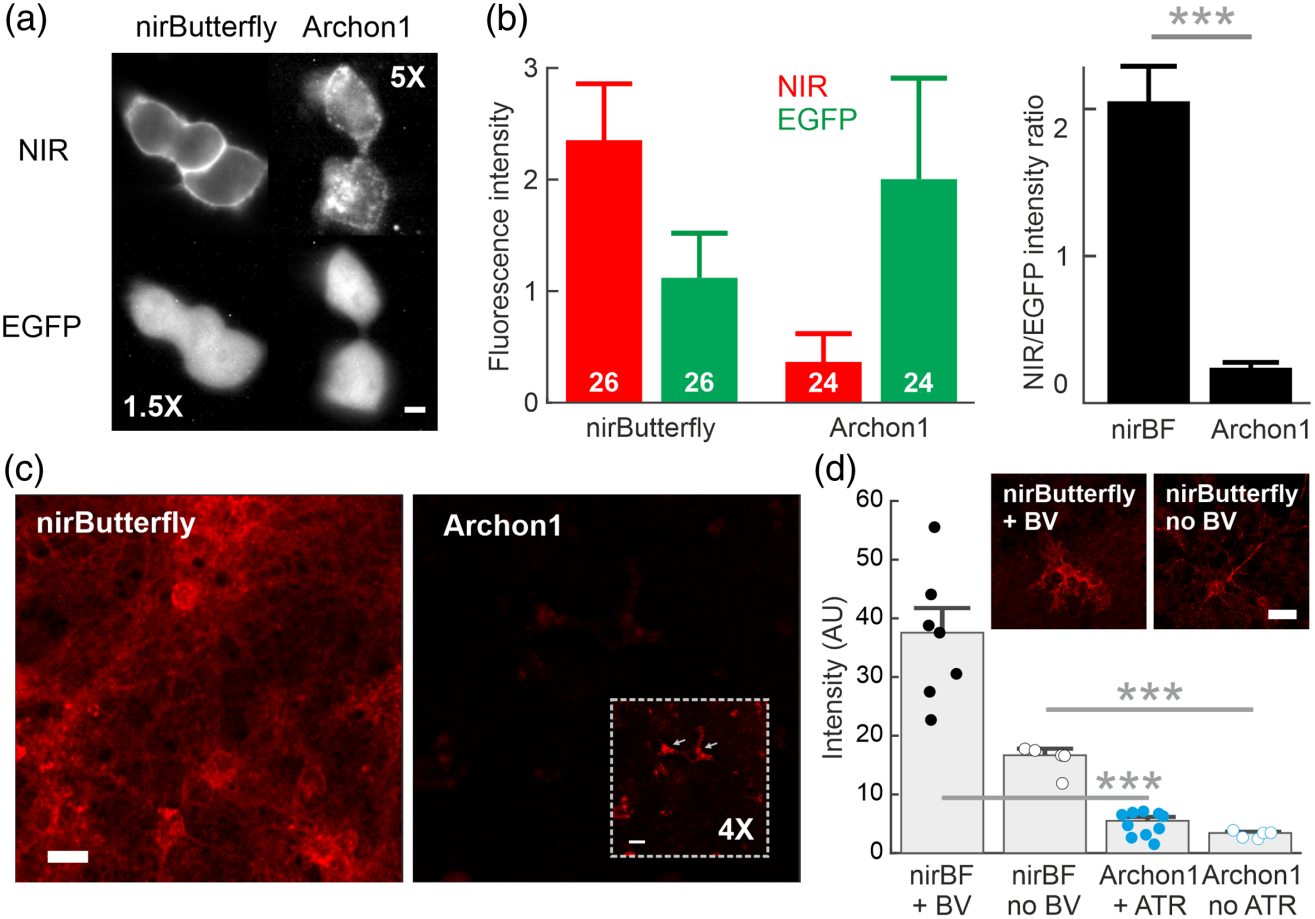
Brightness comparison between nirButterfly and Archon1 in HEK cells and cultured neurons. (a) Representative images of HEK293 cells co-expressing the GEVI (NIR channel, top) and EGFP (below) after being side-by-side transfected with pCAG-nirButterfly-IRES-EGFP (left) or pCAG-Archon1-IRES-EGFP (right). For visualization, image gain is enhanced by 1.5× for the EGFP channel of nirButterfly and 5× for the near-infrared channel of Archon1. Scale bar=10  μm (b) Absolute fluorescence intensities (red-green bars) in the near-infrared and EGFP channels for nirButterfly and Archon1 (N=26 FOVs for nirButterfly; 24 for Archon1). EGFP-normalized fluorescence intensities (black bars). (c) Cultured neurons were side-by-side transduced with AAV1.CaMK2A-nirButterfly or AAV1.CaMK2A-Archon1. Images are shown at the same display gain, both without supplemental chromophores. The Archon1 inset shows the same FOV with the image gain 4× enhanced. Arrows indicate expression aggregates. Scale bar=20  μm. (d) Absolute fluorescence intensities for cultures transduced with nirButterfly (N=10 FOVs for nirButterfly with biliverdin; 5 for nirButterfly without biliverdin) and Archon1 (N=10 for Archon1 with all-trans-retinal; N=5 for Archon1 without all-trans-retinal), both with and without supplements of their respective chromophores (biliverdin for nirButterfly, and all-trans-retinal for Archon1). Data presented in median ± SEM; *** = p<0.001; individual data points shown for N=<10.

The brightness of the two GEVIs was first compared in cultured HEK293T cells. We generated bicistronic plasmids encoding either nirButterfly or Archon1 (each GEVI equipped with a cytosolic EGFP tag on the same plasmid backbone) and performed side-by-side transfection into cultured HEK293T cells [Fig. 2(a)]. We used the cytosolic EGFP expression to normalize across other factors that may introduce variability in total detected brightness, including size variability of the expressing cells and variation in GEVI protein expression levels.

We observed an approximately 1.5-fold higher fluorescence intensity of cytosolic EGFP expression in HEK293T cell cultures transfected with Archon1-IRES-EGFP than nirButterfly-IRES-EGFP [Fig. 2(b), red-green bars]. This could be due to the larger molecular size of nirButterfly than Archon1, which could potentially lead to decreased expression levels. We observed membrane-targeted expression of nirButterfly in the transfected HEK cells, but cytosolic aggregates were typically observed in HEK293T cells expressing Archon1 [Fig. 2(a)]. Such Archon1 aggregates have also been reported previously.^25^^–^^27^ After normalization on the EGFP fluorescence level, we observed on average sevenfold higher fluorescence intensity from nirButterfly versus Archon1 [N=26 FOVs for nirButterfly, 24 for Archon1; p<0.001, two-sample t-test; Fig. 2(b), black bars].

Next, we compared GEVI brightness in cultured primary neurons as they more closely model the conditions for neurophysiological applications. The transfection of plasmids into cultured neurons often has very low transfection efficiency; hence here we used AAV transduction to express the GEVIs. We generated virus plasmids encoding either nirButterfly or Archon1 using an identical virus plasmid backbone. Neuronal cultures of similar seeding density were transduced side-by-side with AAV1.CaMK2A-nirButterfly or AAV1.CaMK2A-Archon1 at similar virus titers [Fig. 2(c)]. The size of the nirButterfly gene is close to the maximal AAV packaging size; therefore, we could not introduce an additional EGFP tag into the AAV cargo plasmid to perform the normalization step as described in Figs. 2(a) and 2(b). Instead, here we measured the absolute fluorescence intensity of the transduced cultures, and similar to HEK293T cell cultures [Figs. 2(a) and 2(b)], in neurons [Fig. 2(c)] we observed an approximately sixfold higher fluorescence intensity from nirButterfly than Archon1, both with and without the additional supplements of their respective chromophores [biliverdin for nirButterfly, *all-trans*-retinal for Archon1; both are present naturally in the mammalian brain; N=10 FOVs for with chromophore supplements, p<0.001, two-sample t-test; and N=5 FOVs for without chromophore supplements, p<0.001, two-sample t-test; Fig. 2(d)].

**Fig. 3:**
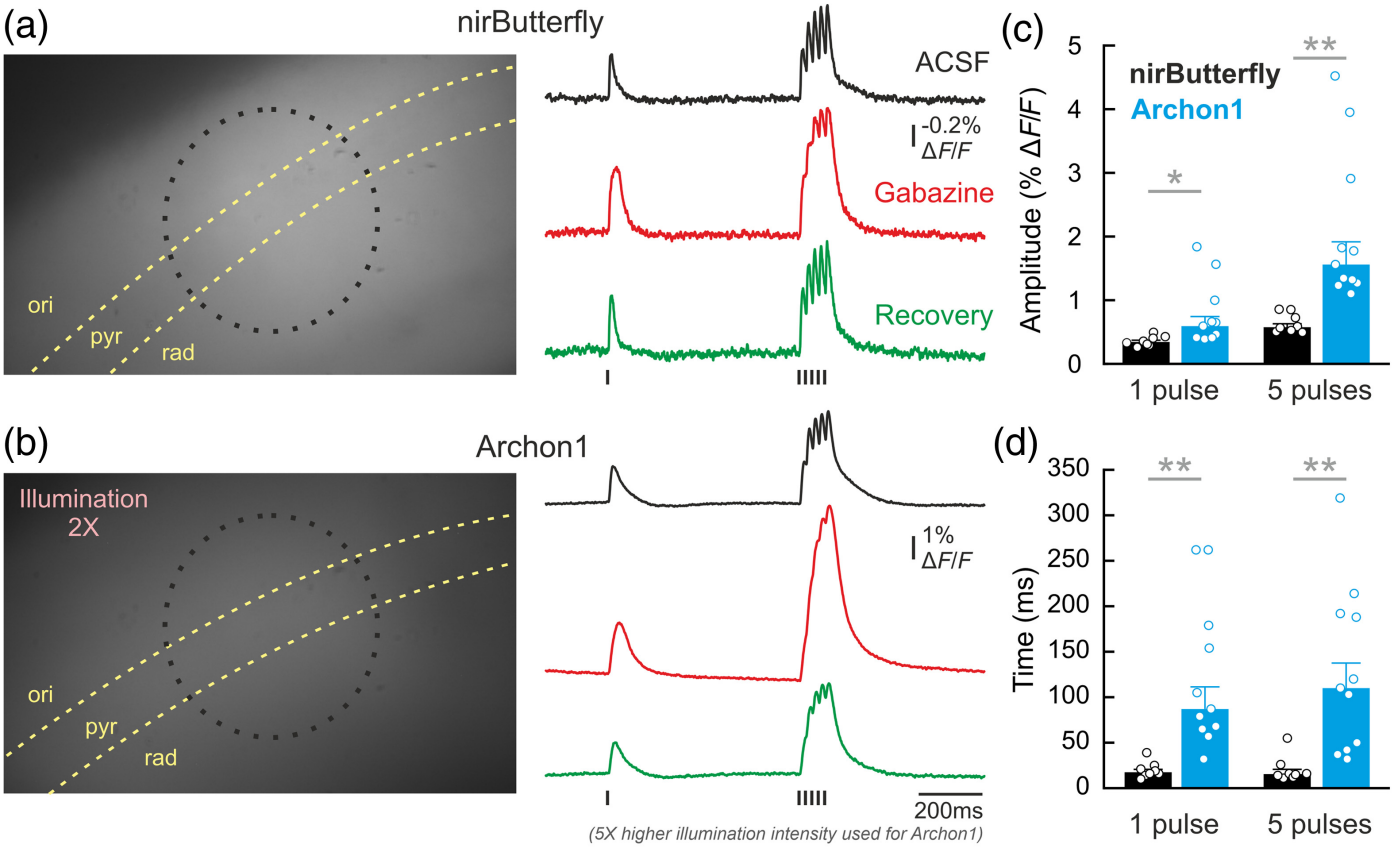
GEVI sensitivity and kinetic comparisons in acute brain slices. (a) (left) AAV-transduced nirButterfly expression in hippocampal pyramidal neurons, and (right) representative population response in CA3 following Schaffer collateral stimulation under standard ACSF, Gabazine-ACSF, and post-Gabazine recovery conditions (10-trial average). The dotted circle outlines the region of photon sampling for the photodiodes. The position of the stimulation electrode was outside the imaging field of view. (b) Same as in (a) for Archon1. Traces were acquired with five times higher illumination intensity. (c) GEVI sensitivity was measured as the population peak response amplitude normalized to baseline fluorescence for following 1-pulse (p=0.024, two-sample t-test with unequal variance) and five-pulse synaptic stimulation (p=0.002, two-sample t-test with unequal variance). (d) GEVI kinetic properties are measured as the decay time constants following 1-pulse (p=0.002, two-sample t-test with unequal variance) and 5-pulse synaptic stimulation (p=0.003, two-sample t-test with unequal variance). nirButterfly: N=8 slices from 3 mice; Archon1: N=11 slices from 3 mice. Data presented in median ± SEM; * = p<0.05. ** = p<0.01.

Then, we transduced pyramidal neurons in the hippocampus of wild-type mice using AAV1.CaMK2A-nirButterfly or AAV1.CaMK2A-Archon1 at similar virus titers. After 3 weeks of expression, we made coronal brain slice preparations and imaged them under epifluorescence imaging conditions [Figs. 3(a) and 3(b), left]. Fluorescence was observed from the cell body in the hippocampal stratum pyramidale and from the intermingled processes in the oriens and radiatum. For slices transduced with AAVs delivering Archon1, approximately five times higher illumination intensity was needed to achieve near comparable fluorescence intensities for functional imaging [Fig. 2(d)].

### 3.2 Archon1 Displays Higher Sensitivity but Slower Kinetics than nirButterfly

Next, we wanted to compare the functional performance of these two GEVIs in brain slices. Here, instead of single cell-level comparisons (Fig. 2), we used epifluorescence population imaging to effectively average across two cardinal factors that are notorious for affecting indicator performance: cell size variability and indicator expression variability; both factors strongly influencing the signal-to-noise ratio (SNR) in voltage imaging traces.

We stimulated Schaffer collaterals that project from CA3 to CA1 and imaged the population responses in CA1 using a photodiode that samples over the region of interest at 10 kHz. Only the donor channel (676/37  nm) was recorded for nirButterfly. Given that the emission detection used in our epifluorescence imaging setup transmits a comparable fraction of the emitted fluorescence of both nirButterfly and Archon1 [Figs. 1(b) and 1(d)], we used the identical optical configuration to measure the functional emission of Archon1.

Both GEVIs reported population optical signals in CA1 following single pulse Schaffer collateral stimulation and five pulses at 50 Hz, with a good SNR [Figs. 3(a) and 3(b), right]. With the addition of the ${\mathrm{GABA}}_{A}$ receptor antagonist gabazine to the perfusate, these population voltage responses increased in amplitude when using both GEVIs, and the response decay rate slowed down, resulting in fused responses to the five pulses. After recovery from gabazine, the responses became shorter-lasting again and more separated in the case of the 5-pulse stimulation.

We also performed population level imaging experiments of synaptic stimulation with the GEVIs being expressed in the cortex. We observed similar optically reported response amplitude for both indicators and therefore pooled the functional data. Quantifications of these population responses were used to assess the sensitivity and kinetics of the GEVI performances in mouse brain tissue. For sensitivity, the absolute peak amplitude for Archon1 was approximately twofold larger than nirButterfly [Fig. 3(c)] for both responses to single pulse synaptic stimulation (Archon1: 0.59±0.15% ΔF/F, nirButterfly: 0.34±0.03% ΔF/F, N=11 slices from 3 mice for Archon1 and 8 slices from 3 mice for nirButterfly, median ± SEM) and 5-pulse stimulation (Archon1: 1.56±0.36% ΔF/F, nirButterfly: 0.58±0.05% ΔF/F, N=11 and 8, respectively, median ± SEM).

We also quantified the decay time constant of these responses as a kinetics comparison between the two indicators. The response decay for Archon1 was approximately four times slower than that of nirButterfly [Fig. 3(d)], for both single pulse (Archon1: 87.00±24.37  ms, nirButterfly: 17.50±3.15  ms; Tau of a single exponential fit, N=11 and 8, respectively, median ± SEM) and 5-pulse stimulation (Archon1: 110.00±27.56  ms, nirButterfly: 15.50±1.11  ms; Tau of a single exponential fit, N=11 and 8, respectively, median ± SEM). This slower response decay has been noted in previous work (res) and may also contribute to the larger peak amplitude of Archon1 signals observed at sampling rates <500  Hz. A slower decay also can cause larger GEVI signal amplitudes with repetitive stimulation. These results were surprising given the faster kinetics generally declared for opsin-based GEVIs, but the potential mechanisms underlying this feature of Archon1 require further elucidation.

## 4 Discussion

Optical imaging in the near-infrared wavelength range offers many advantages for monitoring *in vivo* neuronal activity in the mammalian brain. In addition to deeper tissue penetration and reduced tissue scattering, near-infrared imaging in the brain offers many additional powerful capabilities, including the reduced optical influence of hemodynamic activities,^21^^,^^28^^,^^29^ having the ability to be used simultaneously with blue-shifted optogenetic actuators within the sample preparation for all-optical electrophysiology experiments,^14^^,^^15^^,^^23^^,^^30^ and it can be combined with activity indicators of other spectral properties for multicolor functional imaging.

Parallel to the development of nirButterfly, efforts in molecular fine-tuning of opsin-based GEVIs generated Archon variants. With the increasing expansion of the GEVI toolbox, a question from most tool users is “which GEVI to choose for my experiments.” Here, we were interested in comparing the performance of nirButterfly and Archon1, not only for the scientific interest of molecular tool development but also in selecting a better-performing GEVI for addressing physiological questions.

Compared with Archon1, nirButterfly offers much higher brightness under similar imaging conditions in both cultured GEVI-expressing HEK293T cells and GEVI-expressing primary neurons (Fig. 2). In all cultures examined, we observed better membrane-targeted GEVI expression in cells expressing nirButterfly than Archon1.

Despite needing higher illumination intensities, Archon1 appears to have significantly higher sensitivity (i.e., larger percentage ΔF/F) in population imaging experiments in acute brain slices compared with nirButterfly [Figs. 3(b) and 3(c)], and this higher sensitivity can be attractive in some experimental situations. Opsin-based GEVIs offer sub-millisecond resolution kinetic properties in single-cell recordings.^15^ In this study, in population voltage imaging without single-cell resolution, we were surprised to see the slower kinetic properties of Archon1 compared with nirButterfly, measured under identical conditions [Fig. 3(d)]. This slower kinetics of Archon1 potentially contributes to its larger ΔF/F, but the mechanistic basis of this observation requires further exploration.

Achieving a good SNR is a critical issue when selecting a particular GEVI for a particular type of experiment. Photophysical theory states that the SNR depends on the expression level, molecular fluorescence quantum yield, and sensitivity.^31^ Neither GEVI characterized here surpasses in all three of these key parameters. The SNR is often taken alone as an indicator of measurement quality, but it may not serve as the best parameter to compare indicators, and the SNR would at least need to be normalized on the bleaching-time measurement to achieve a fair comparison of the suitability (feasible recording times) between two or more fluorescent indicators. For example, increasing an excitation intensity by a factor of 100 would increase the SNR by a factor of 10. However, this will not make a “better” or “more practical” indicator because it would limit the time during which informative physiological measurements can be obtained. For this reason, a dim GEVIs may be disadvantageous in terms of tissue heating and tissue damage by strong excitation light. On the other hand, the experimental design may require just one or two short-lasting optical traces from the same region of interest. In that case, when tissue damage is not a concern, a dim but sensitive GEVI may be advantageous. Taken together, there is no unequivocal preference for one of the two GEVIs characterized here.

Our side-by-side characterization presented here aims to yield essential information for future *in vitro* and *ex vivo* experimental designs. Here, our experiments were performed under the same optical configuration to avoid confusion of the configuration-specific and indicator-specific characteristics. An increase of excitation intensities, using optics with increased photon sampling efficacies and detectors with higher photon quantum yield, would all have predictable effects on the SNR, and these effects would scale—within reasonable limits in the shot-noise limited regime—in a similar way for both indicators. The impacts of the illumination power on the optical signal quality and optical indicator photostability are—again within reasonable limits in a shot noise limited regime—governed by biophysical principles: the SNR is proportional to the square root of the fluorescence intensity, which is proportional to the excitation intensity. Similarly, photobleaching is also proportional to the excitation intensity. Although the *ex vivo* (brain slice) measurements offer a strong estimation for a GEVI’s relative performance *in vivo*,^32^ additional parameters will need to be considered and further optimized during *in vivo* applications, such as molecular tools for optimal expression^19^ and experimental hardware configurations,^30^ to achieve the most ideal recording quality.

## Data Availability

Further information and requests for resources and reagents should be directed to the lead contact (tknopfel@knopfel-lab.net). Raw data reported in this paper will be shared by the lead contact upon request. This paper does not report original code.
